# The Parkinsonian Personality: More Than Just a “Trait”

**DOI:** 10.3389/fneur.2018.01191

**Published:** 2019-01-15

**Authors:** Antonina Luca, Alessandra Nicoletti, Giovanni Mostile, Mario Zappia

**Affiliations:** Dipartimento di Scienze Mediche, Chirurgiche e Tecnologie Avanzate “G.F. Ingrassia”, Università degli Studi di Catania, Catania, Italy

**Keywords:** personality disorders, obsessive-compulsive personality disorder, Parkinson's disease, temperament, harm avoidance

Since 1913 patients with Parkinson's disease (PD) have been described as particularly industrious, devoted to hard work, inflexible, punctual, cautious, and moralist (1). These psychological characteristics have been so constantly reported that the concept of “*Parkinsonian personality*” emerged. In this regards, in the last few years PD patients have been evaluated according to several models of personality assessment (2), with the Big Five Model (BFM) (3) and the Cloninger's Psychobiological Model (CPM) (4) as the most used. Studies following the BFM reported that PD patients presented high levels of Neuroticism and low levels of both Openness and Extraversion (5, 6), while studies using the CPM described the temperament of PD patients as characterized by low Novelty Seeking (NS) and high Harm Avoidance (HA) (2, 7, 8). As a matter of fact, the high HA could be responsible for the Parkinsonians' tendency to be cautious, fearful, pessimistic and shy, while the low levels of NS could account for the tendency to be unsocial, frugal and orderly. Under different points of view, the “Parkinsonian personality,” as it has been consistently reported in literature (7, 9), shares several clinical features with the obsessive compulsive personality disorder (OCPeD) as classified in the Diagnostic and Statistical Manual for Mental Disorders (DSM) (10).

The OCPeD is defined as a “chronic, pervasive, maladaptive pattern of preoccupation with orderliness, perfectionism, mental and interpersonal control at the expense of flexibility, openness, and efficiency” (10). In the general population, it is the most common personality disorder with a lifetime prevalence reaching the 9.3% (11). Classically considered as stable over time, an increasing number of observations allow to hypothesize that the clinical presentation of OCPeD is less stable than originally assumed, being possible to detect the occurrence, attenuation, or relapse of obsessive symptoms across the life-time (12, 13). While the correspondence between the presence of high HA and low NS levels and OCPeD has been investigated in the general population over the years (14–16), no studies on the correspondence between these temperament traits, configuring the parkinsonian personality, and OCPeD have been conducted in PD patients.

However, studying the “Parkinsonian personality” according to the DSM diagnostic criteria could be more useful in terms of clinical practice, since it would allow to reach a diagnosis of personality disorder rather than merely describe temperamental traits.

On the contrary, nowadays there is a limited amount of studies using the DSM criteria.

In particular, we have carried out a study with the aim of investigating the presence of personality disorders in PD patients and controls, reporting a significantly higher frequency of OCPeD among PD patients (respectively 40 vs. 10%) (17). In agreement with some literature evidences that reported a quite common prevalence of OCPeD in the elderly (18), the frequency of OCPeD was higher among PD patients aged 60 years and above (17). These findings suggest that even among PD patients, OCPeD are probably not so stable in the life-span, worsening during senescence (11). Furthermore, the high frequency of OCPeD was also confirmed in *de novo* PD patients with a short disease duration, thus supporting the hypothesis that this personality disorder could be considered as an early non-motor manifestation of PD, being present regardless disease duration, motor impairment or dopaminergic treatment (19). Regarding other movement disorders, a high prevalence of OCPeD has been also found among patients with Progressive Supranuclear Palsy (PSP) but not among those with multiple system atrophy or essential tremor (20). PSP is an atypical parkinsonism characterized by perseverations, rigid perfectionism, restricted affectivity and executive function deficit (21). Actually, from a pathophysiological point of view, the high prevalence of OCPeD both in PD and in PSP patients, could be linked to a common frontostriatal circuit dysfunction (22); indeed, it may be useful to deepen the knowledge regarding the possible association between personality changes and executive dysfunctions. Also the latters, in fact, essential for everyday decision-making (such as planning, monitoring, manipulating information and attention), are mostly modulated by dorsal fronto-striatal loops (23). It has been reported that more than 30% of PD patients present, even at an early stage of the disease, a cognitive condition “intermediate” between normal cognition and dementia, primarily marked by attention and executive functions impairment (24).

Few studies have investigated the possible association between the “parkinsonian personality” and executive dysfunction reporting controversial results, also considering methodological differences used by different studies (6, 25, 26). In particular, Volpato et al. (6), assessing the personality according to the BFM, reported a certain correlation between specific personality traits (emotional stability and openness to experience) and executive functions. Luca et al. (25), assessing the personality according to the CPM, reported a strong association between high HA score and executive dysfunctions, while Koerts et al. (26) did not.

Unfortunately, while in the general population the association between OCPeD and executive dysfunction has been elucidated (27, 28), no literature data on PD patients are now available. However, due to the aforementioned close correspondence between high HA score and OCPeD, it is not unlikely that the reported association between high HA and executive dysfunction could be just an epiphenomenon, reflecting the well-known association between OCPeD and executive dysfunctions. As a matter of fact, the corticostriatal circuit dysfunction (orbitofrontal cortex, medial prefrontal cortex and striatum) could represent the pathophysiological explanation of both cognitive and behavioral inflexibility characterizing OCPeD (29).

In any case, regardless the co-occurrence of executive function deficits, the assessment of parkinsonian personality through a clinical interview evaluating the possible presence of an OCPeD should be encouraged for different reasons. First of all, as previously mentioned, the OCPeD is not just a “temperament trait,” but a nosographically defined clinical entity that can be diagnosed according to worldwide accepted criteria (10). Moreover, OCPeD frequently occurs in association with other psychiatric disturbances, such as anxiety disorders, depression, alcohol or drug dependence, hypochondriasis and obsessive-compulsive spectrum disorders (30), pathological gambling, and internet addiction, above all (31, 32).

In the light of what has been said, the assessment of OCPeD in PD patients could be useful also in the stratification of frailty allowing neurologists to identify those patients at risk of impulse control disorders or cognitive decline. Finally, severe OCPeD can benefit from antidepressants and/or psychotherapeutic interventions (33–35).

In conclusion, the assessment of personality disorders in PD patients according to the DSM would impact clinical practice, guaranteeing the formulation of a clear diagnosis, thus enabling the clinicians to take the right therapeutic path, in line with a patient-centered point of view.

## Author Contributions

AL, AN, GM, and MZ: conception; AL and AN: writing of the first draft; All authors: review and critique the manuscript.

### Conflict of Interest Statement

The authors declare that the research was conducted in the absence of any commercial or financial relationships that could be construed as a potential conflict of interest.

## References

[1] Camp CD Paralysis agitans, multiple sclerosis and their treatment. In: Modern Treatment of Nervous and Mental Disease. Vol. 2, Philadelphia (1913). p. 651–67.

[2] Cerasa A Re-examining the Parkinsonian personality hypothesis: a systematic review. Pers Individ Differ. (2018) 130:41–50. 10.1016/j.paid.2018.03.045

[3] Costa PT McCrae RR Personality disorders and the five-factor model of personality. J Pers Disord. (1990) 4:362–71. 10.1521/pedi.1990.4.4.362

[4] Cloninger CR A systematic method for clinical description and classification of personality variables. Arch Gen Psychiatry (1987) 44:573–88. 10.1001/archpsyc.1987.01800180093014 3579504

[5] Baig F Lawton MA Rolinski M Ruffmann C Klein JC Nithi K. Personality and addictive behaviours in early Parkinson's disease and REM sleep behaviour disorder. Parkinsonism Relat Disord. (2017) 37:72–8. 10.1016/j.parkreldis.2017.01.017 28173973 PMC5380654

[6] Volpato C Signorini M Meneghello F Semenza C. Cognitive and personality features in Parkinson disease: 2 sides of the same coin? Cogn Behav Neurol. (2009) 22:258–63. 10.1097/WNN.0b013e3181c12c63 19996879

[7] Menza M. The personality associated with Parkinson's disease. Curr Psychiatry Rep. (2000) 2:421–6. 11122991 10.1007/s11920-000-0027-1

[8] Santangelo G Garramone F Baiano C D'Iorio A Piscopo F Raimo S. Personality and Parkinson's disease: a meta-analysis. Parkinsonism Relat Disord. (2018) 49:67–74. 10.1016/j.parkreldis.2018.01.013 29358028

[9] Ishihara L Brayne C. What is the evidence for a premorbid parkinsonian personality? A systematic review. Mov Disord. (2006) 21:1066–72. 10.1002/mds.20980 16755553

[10] American Psychiatric Association Diagnostic and Statistical Manual of Mental Disorders. 5th ed. (DSM-IV). Washington, DC: American Psychiatric Press (2013).

[11] Torgersen S Kringlen E Cramer V. The prevalence of personality disorders in a community sample. Arch Gen Psychiatry (2001) 58:590–6. 10.1001/archpsyc.58.6.590 11386989

[12] Amad A Geoffroy PA Vaiva G Thomas P. Personality and personality disorders in the elderly: diagnostic, course and management. Encephale (2012) 39:374–82. 10.1016/j.encep.2012.08.006 23095604

[13] Ullrich S Coid J The age distributions of self-reported personality disorder traits in a household population. J Personal Disord. (2009) 23:187–200. 10.1521/pedi.2009.23.2.187 19379095

[14] Kantojärvi L Miettunen J Veijola J Läksy K Karvonen JT Ekelund J. Temperament profiles in personality disorders among a young adult population. Nord J Psychiatry (2008) 62:423–30. 10.1080/08039480801959224 18839387

[15] Casey JE Joyce PR. Personality disorder and the temperament and character inventory in the elderly. Acta Psychiatr Scand. (1999) 100:302–8. 10.1111/j.1600-0447.1999.tb10865.x 10510700

[16] Skodol AE McGrath PJ Oldham JM. Relationship between the tridimensional personality questionnaire and DSMIII-personality traits. Am J Psychiatry (1994) 151:274–6. 10.1176/ajp.151.2.274 8296904

[17] Nicoletti A Luca A Raciti L Contrafatto D Bruno E Dibilio V. Obsessive compulsive personality disorder and Parkinson's disease. PLoS ONE (2013) 8:e54822. 10.1371/journal.pone.0054822 23359811 PMC3554639

[18] Grant JE Mooney ME Kushner MG. Prevalence, correlates, and comorbidity of DSM-IV obsessive-compulsive personality disorder: results from the National Epidemiologic Survey on alcohol and related conditions. J Psychiatr Res. (2012) 46:469–75. 10.1016/j.jpsychires.2012.01.009 22257387

[19] Nicoletti A Luca A Luca M Mostile G Sciacca G Petralia A. Obsessive-compulsive personality disorder in drug-naïve Parkinson's disease patients. J Neurol. (2015) 262:485–6. 10.1007/s00415-014-7617-z 25522699

[20] Nicoletti A Luca A Luca M Donzuso G Mostile G Raciti L. Obsessive compulsive personality disorder in progressive supranuclear palsy, multiple system atrophy and essential tremor. Parkinsonism Relat Disord. (2016) 30:36–9. 10.1016/j.parkreldis.2016.06.015 27364040

[21] Schrag A Sheikh S Quinn NP Lees AJ Selai AJ Mathias C. A comparison of depression, anxiety, and health status in patients with progressive supranuclear palsy and multiple system atrophy. Mov Disord. (2010) 25:1077–81. 10.1002/mds.22794 20535826

[22] Fineberg NA Potenza MN Chamberlain SR Berlin HA Menzies L Bechara A. Probing compulsive and impulsive behaviors, from animal models to endophenotypes: a narrative review. Neuropsychopharmacology (2010) 35:591–604. 10.1038/npp.2009.185 19940844 PMC3055606

[23] Bentivoglio AR Baldonero E Ricciardi L De Nigris F Daniele A. Neuropsychological features of patients with Parkinson's disease and impulse control disorders. Neurol Sci. (2013) 34:1207–13. 10.1007/s10072-012-1224-5 23161255

[24] Monastero R Cicero CE Roberta B Davì M Luca A Restivo V. Mild cognitive impairment in Parkinson's disease: the Parkinson's disease cognitive study. J Neurol. (2018) 265:1050–8. 10.1007/s00415-018-8800-4 29478221

[25] Luca A Nicoletti A Mostile G Sciacca G Dibilio V Cicero CE. Temperament traits and executive functions in Parkinson's disease. Neurosci Lett. (2018) 684:25–8. 10.1016/j.neulet.2018.06.040 29940327

[26] Koerts J Tucha L Leenders KL Tucha O. Neuropsychological and emotional correlates of personality traits in Parkinson's disease. Behav Neurol. (2013) 27:567–74. 10.3233/BEN-129017 23242356 PMC5214952

[27] Garcia-Villamisar D Dattilo J Garcia-Martinez M. Executive functioning in people with personality disorders. Curr Opin Psychiatry (2017) 30:36–44. 10.1097/YCO.0000000000000299 27798484

[28] Aycicegi-Dinn A Dinn WM Caldwell-Harris CL. Obsessive-compulsive personality traits: compensatory response to executive function deficit? Int J Neurosci. (2009) 119:600–8. 10.1080/00207450802543783 19229723

[29] Figee M Pattij T Willuhn I Luigjes J van den Brink W Goudriaan A. Compulsivity in obsessive-compulsive disorder and addictions. Eur Neuropsychopharmacol. (2016) 26:856–68. 10.1016/j.euroneuro.2015.12.003 26774279

[30] Diedrich A Voderholzer U. Obsessive–compulsive personality disorder: a current review. Curr Psychiatry Rep. (2015) 17:2. 10.1007/s11920-014-0547-8 25617042

[31] Chamberlain SR Redden SA Stein DJ Lochner C Grant JE. Impact of obsessive-compulsive personality disorder symptoms in internet users. Ann Clin Psychiatry (2017) 29:173–81. 28738097 PMC5540167

[32] Medeiros GC Grant JE. Gambling disorder and obsessive–compulsive personality disorder: a frequent but understudied comorbidity. J Behav Addict. (2018) 7:366–74. 10.1556/2006.7.2018.50 29936850 PMC6174606

[33] Greve KW Adams D. Treatment of features of obsessive-compulsive personality disorder using carbamazepine. Psychiatry Clin Neurosci. (2002) 56:207–8. 10.1046/j.1440-1819.2002.00946.x 11952927

[34] Ekselius L von Knorring L Personality disorder comorbidity with major depression and response to treatment with sertraline or citalopram. Int Clin Psychopharmacol. (1998) 13:205–11. 10.1097/00004850-199809000-00003 9817625

[35] Bamelis LL Evers SM Spinhoven P Arntz A. Results of a multicenter randomized controlled trial of the clinical effectiveness of schema therapy for personality disorders. Am J Psychiatry (2014) 171:305–22. 10.1176/appi.ajp.2013.12040518 24322378

